# Association of Adiponectin Receptors with Metabolic and Immune Homeostasis Parameters in Colorectal Cancer: In Silico Analysis and Observational Findings

**DOI:** 10.3390/ijerph192214995

**Published:** 2022-11-14

**Authors:** Marija Mihajlović, Ana Ninić, Marija Ostojić, Miron Sopić, Aleksandra Stefanović, Jelena Vekić, Tamara Antonić, Dejan Zeljković, Bratislav Trifunović, Vesna Spasojević-Kalimanovska, Nataša Bogavac Stanojević, Ivan Jančić, Aleksandra Zeljković

**Affiliations:** 1 Department of Medical Biochemistry, Faculty of Pharmacy, University of Belgrade, 11000 Belgrade, Serbia; 2 Department of Experimental Oncology, Institute for Oncology and Radiology of Serbia, 11000 Belgrade, Serbia; 3 Clinic of General Surgery, Military Medical Academy, 11000 Belgrade, Serbia; 4 Faculty of Medicine, Military Medical Academy, University of Defense, 11000 Belgrade, Serbia; 5 Department of Microbiology and Immunology, Faculty of Pharmacy, University of Belgrade, 11000 Belgrade, Serbia

**Keywords:** CRC, ADIPORs, TNF-α, SNP, metabolism, immunity

## Abstract

Adiponectin (ADIPOQ) as both a regulator of metabolic homeostasis and a protein involved in immune response might be of particular interest to contemporary laboratory medicine, especially in terms of minimally invasive diagnostics. The diverse roles of ADIPOQ with regard to the immune and metabolic aspects of colorectal carcinogenesis have been proposed. However, the expression of its receptors ADIPOR1 and ADIPOR2 is scarcely explored in peripheral blood mononuclear cells (PBMCs). Moreover, ADIPORs’ relationships with the immune response mediator TNF-α have not been previously investigated in the PBMCs of CRC patients. This study used both in silico and observational case–control analyses with the aim of exploring the association of ADIPOR gene expression and ADIPOQ single nucleotide polymorphisms (SNPs) with the inflammatory marker TNF-α and lipid status parameters in patients with CRC. Publicly available transcriptomic datasets (GSE47756, GSE44076) obtained from analyses of monocytes and CRC tissue samples were employed for the in silico evaluation of ADIPORs’ specific genetic traits. GSE47756 and GSE44076 datasets were processed with GSEA software to provide a genetic fingertip of different signaling pathways associated with ADIPORs’ mRNA levels. The case–control aspect of the study included the PBMC samples of 73 patients diagnosed with CRC and 80 healthy volunteers. The PCR method was carried out for the PBMC gene expression analysis (ADIPOR1, ADIPOR2, TNF-α mRNA levels) and for the subjects’ genotyping (ADIPOQ rs266729, ADIPOR1 rs7539542). GSEA showed significant associations of ADIPOR mRNA expression with gene sets related to metabolic and immune homeostasis in both datasets. The case–control study revealed the association of ADIPOR1 rs7539542 with reduced lipid status parameters in CRC. In addition, PBMC ADIPOR1 mRNA levels decreased in CRC (*p* < 0.001), whereas ADIPOR2 mRNA did not differ between the groups (*p* = 0.442). A reduction in PBMC TNF-α mRNA levels was noted in CRC (*p* < 0.05). Our results indicate that ADIPOR1 and ADIPOR2 play a significant role in the alteration of both metabolic and immune homeostasis during the progression of CRC. For the first time, ADIPOR1 is shown to be a specific receptor for mediating ADIPOQ’s effects in the PBMCs of CRC patients.

## 1. Introduction

Colorectal cancer (CRC) is a widespread disease and the second leading cause of cancer-related deaths worldwide [1]. Thus, there is a constant need for more advanced diagnostic and prognostic tools in order to perform the rapid, accurate and cost-effective detection of CRC onset. One existing innovation in personalized laboratory medicine is multiplex testing [2]. However, despite all the efforts so far, studies evaluating different biomarker coherences are still lacking. Nevertheless, this analysis could complement the intensive search for appropriate multiplex biomarkers, which might be especially beneficial in cancer.

Studies of multistep cancer development shed light on the role of the tumor microenvironment, and the participation of immune cells in both disease worsening and improvement has been underlined [3]. The heterogeneity of immune system irregularities during the pathogenesis of CRC represents a valuable research field for developing novel immunomodulatory therapeutic approaches and diagnostic tests [3]. Immunity is therefore an area with many possibilities for the advancement of the multiplex approach in diagnostics [4]. Moreover, previous studies implicate the importance of adipocytokine signaling pathways that interfere with the cascades induced by immune response mediators [5]. The possible cross-talk of these two groups of molecules may result in pleiotropic effects during cancer development. Adiponectin (ADIPOQ) should be singled out, since this adipocytokine is both a regulator of metabolic homeostasis and a protein tied in with the immune response [5,6]. Namely, ADIPOQ is recognized as a protective, anti-inflammatory molecule, and reiterated evidence has implied its antineoplastic properties [7]. However, it should be noted that although ADIPOQ is mainly regarded as an anti-proliferative and anti-inflammatory molecule, conflicting observations were also reported [8]. ADIPOQ’s purpose in cancer development therefore still remains unresolved.

In order to adequately address ADIPOQ’s numerous roles in cancer and immunity, the research focus has shifted toward its receptors: ADIPOR1, ADIPOR2 and T-cadherin [9]. The contribution of ADIPOR1 to mediating the attenuation of nuclear factor κB (NF-κB) pro-inflammatory signaling pathway in macrophages has already been established. By contrast, a presumed role of ADIPOR2 is the inducement of macrophage polarization to the M2 subpopulation [10]. The significance of ADIPOQ and ADIPOR genetic variants has also been underlined by multiple studies, which pointed to an association of specific single nucleotide polymorphisms (SNPs) with CRC development [11]. Although important for colorectal carcinogenesis, the ADIPORs’ fates are reportedly ambiguous. For instance, Byeon et al. [12] found an inverse correlation between the stage of the disease and ADIPOR1/ADIPOR2 immunostaining, while Williams et al. [13] showed that these receptors have no association with disease severity. By contrast, immunostaining was mainly positive in most cases [13]. Considering that previous research yielded inconsistent results, the importance of ADIPOQ–ADIPOR interaction for CRC development, both at the protein and gene level, should be further inspected. Notably, these biomolecules have been scarcely investigated in peripheral blood mononuclear cells (PBMCs). However, modern diagnostics tend to rely on minimally invasive procedures; thus, biomarkers which could be detected in venous blood samples are highly desirable.

In addition to metabolic and immune homeostasis disturbances, chronic inflammation accompanies cancer progression [14]. Among many pro-inflammatory cytokines, the role of tumor necrosis factor alpha (TNF-α) in colon cancer has been extensively reviewed [15]. Studies revealed that TNF-α binding to a corresponding receptor leads to the successful activation of NF-κB and activator protein 1 (AP-1) signaling cascade, thus inducing prolonged cell survival [16]. On the other hand, TNF-α is recognized as an activator of innate and adaptive immunity, leading to T cell antitumor immune response [17]. Although the function of TNF-α in CRC is not fully elucidated, this cytokine is recognized as being highly relevant to colorectal carcinogenesis [15]. In addition, studies have shown that ADIPOQ structurally resembles TNF-α [9]. Moreover, ADIPOQ has been shown to be responsible for changes in TNF-α production and vice versa [18,19]. Even though there is a body of evidence regarding the association of ADIPOQ and TNF-α, only a few studies have addressed the relationship between this immune mediator and ADIPOQ receptors, especially in cancer. ADIPOQ’s protective effects through a reduced TNF-α inflammatory response via ADIPOR1-dependent activation were previously evaluated in endothelial cells of the umbilical vein; however, this association has scarcely been explored in CRC [20].

Given that metabolic and immune system homeostasis are both disturbed during CRC development [21,22], in this study we intended to investigate the participation of ADIPOQ–ADIPOR interaction in both processes. By using an innovative approach, we aimed to inspect ADIPOR gene expression levels in PBMCs. Cancer, not being an isolated pathological process, affects immune cells. Thus, PBMCs, which encompass the majority of innate and adaptive immune response effectors, represent a reliable biological material. In addition, previous findings named PBMCs as valid surrogate models for genetic analyses.

The in silico part of the study employed two data sets originating from malignant tissue and peripheral blood monocytes (PBM). Such data enabled us to evaluate and compare ADIPORs’ specific genetic imprints from different biological sources. Datasets were also processed with gene set enrichment analysis (GSEA) software to identify different signaling pathways best associated with ADIPOR1 and ADIPOR2 mRNA levels. Finally, subjects were genotyped for polymorphisms of interest, considering that ADIPOQ and ADIPOR1 SNPs can cause the improper functioning of the ADIPOQ axis and have important clinical implications for cancer development.

Given the above, analyses were performed in the following order: Obtained in silico results led us to experimentally explore ADIPOR homeostasis association with immune response biomarker TNF-α, as well as its association with metabolic status representatives—lipid status parameters. The case–control part of the study aimed to analyze differences in ADIPOR1, ADIPOR2 and TNF-α mRNA levels in the PBMCs of patients with CRC and healthy individuals. Significant associations of ADIPOR1 SNP (rs7539542; g.202940846 G > C) with ADIPOR1, ADIPOR2 and TNF-α mRNA levels, as well as with serum lipid concentrations, were evaluated in the CRC group. Analogous analyses were performed for ADIPOQ (rs266729; g.186841685 C > G) polymorphism.

## 2. Materials and Methods

Publicly available transcriptomic datasets GSE47756 [23] and GSE44076 [24] were downloaded from the NCBI Gene Expression Omnibus (GEO) database in pre-processed form with normalized expression values presented on a log2-scale. To eliminate multiple probes, datasets were uploaded to GSEA Broad Institute software [25] to collapse the dataset from probes to symbols using the max probe collapsing mode. These two datasets were analyzed with GSEA software using the Hallmark (h.all.v7.1.symbols.gmt) and Kegg (c2.cp.kegg.v7.1.symbols.gmt) gene sets to find gene expression signatures that best correlate with ADIPOR1 and ADIPOR2 expression using the Pearson correlation with 1000 permutations, and permutation type was set to phenotype. Gene sets that met the false discovery rate lower than the 25% criterion were considered significant [25]. In the GSE44076 dataset, three groups were formed: 50 healthy controls and matched 98 samples of stage II colon tumor tissue and adjacent mucosa. ADIPOR1, ADIPOR2 and TNF expression levels were analyzed between the groups using one-way ANOVA and Dunnett’s multiple comparison test in GraphPad Prism 7 (GraphPad Software, La Jolla, CA, USA). The paired sample t-test was used to eliminate intra-individual variances. At the time of writing this manuscript, there were no available datasets regarding PBMCs’ genetic imprints in CRC; therefore, we analyzed the GSE47756 dataset, which contained information on peripheral blood monocyte (PBM) gene signatures related to CRC progression [23]. Samples were divided into two groups (38 samples from healthy volunteers and 55 from CRC patients) and were analyzed by Student’s t-test in GraphPad Prism 7.

### 2.1. Subjects

This research is part of a larger project which inspected a panel of inflammatory, oxidative and lipidemic risk factors for CRC development and their possible use in disease prediction and diagnosis [26,27]. In the current study, 73 patients were included. These 73 (f/m 24/49) individuals were for the first time diagnosed with CRC at the Clinic for General Surgery of the Military Medical Academy in Belgrade, while 80 (f/m 38/42) healthy volunteers were recruited as the control group during a regular medical check-up at the Medigroup Health Center in Belgrade. Diagnosis of CRC was set based on histopathological examination of the resected tissue. Patients were not previously diagnosed with any other malignant disease nor subjected to any neoadjuvant or hypolipidemic treatment. In addition, an obligatory demand for the study entry was the absence of any other acute or chronic gastrointestinal condition. Therefore, the selection criteria for the control group included the absence of acute or chronic heart, kidney, gut or liver diseases as well as previously or currently diagnosed malignant diseases. In addition, the control group participants were free of any lipid-lowering therapy.

Samples from CRC patients were collected immediately before the surgical procedures, while trained medical technicians collected blood from healthy participants during a medical check-up, abiding by the rule of overnight fasting. After appropriate serum and plasma centrifugation procedures, samples were aliquoted and frozen at −80 °C until further analyses. Routine laboratory testing (total cholesterol-TC, low-density lipoprotein cholesterol-LDL-C, high-density lipoprotein cholesterol -HDL-C, triglycerides -TG) was carried out on an automated analyzer ILAB 300+ (Instrumentation Laboratory, Milan, Italy). Ficoll-Paque^®^ gradient gel was used to isolate PBMCs, followed by immediate addition of TRIzol^TM^ reagent (Invitrogen Life Technologies, Carlsbad, CA, USA) and storage of nucleic acids at −80 °C. After thawing, subsequent isolation of total RNA according to the adapted TRIzol^TM^ manufacturer protocol was performed [28]. Gene expression data are presented as normalized mRNA levels. For the normalization of data, we used beta actin as a housekeeping gene (Hs99999903_m1). Additionally, 200 μL of whole blood was obtained for DNA isolation by employing a commercially available kit for subsequent SNP analysis (GeneJET Whole Blood Genomic DNA Purification Mini Kit; ThermoFisher Scientific, Waltham, MA, USA).

Details of reverse transcription and quantitative polymerase chain reaction (qPCR) are provided elsewhere [27]. TaqMan^®^ reagent-based chemistry was employed for these purposes. Assay ID numbers were as follows: (ADIPOR1: Hs01114951_m1; ADIPOR2: Hs00226105_m1; TNF-α: Hs00174128_m1). Genotyping, reverse transcription and gene expression analysis were performed on 7500 Real-Time PCR System (Applied Biosystems, Foster City, CA, USA). Genotyping of ADIPOR1 rs7539542 (G/C) (Assay ID: C__30041594_10) and ADIPOQ rs266729 (C/G) (Assay ID: C___2412786_10) was conducted according to the reagent manufacturer guidance (TaqMan^®^ SNP Genotyping Assays, Applied Biosystems, Foster City, CA, USA). In addition, CC homozygotes of ADIPOR1 rs7539542 SNP represented one group, while all carriers of G allele constituted the group for comparison (CG + GG). Merging of genotypes was performed due to a small number of GG homozygotes. Analogously, the same groups were formed for ADIPOQ rs266729 analysis.

### 2.2. Case–Control Study Statistical Analysis

The normality of data in groups with more than 50 subjects was estimated by using the Kolmogorov–Smirnov test, while for smaller cohorts, the Shapiro–Wilk test was employed. Normally distributed data are presented as mean ± standard deviation. Data that achieved normal distribution after logarithmic transformation are presented as geometrical mean with 95% CI, while asymmetrically distributed data are shown as medians and interquartile range. The chi-square test was used for categorical data analysis as well as for Hardy–Weinberg equilibrium testing. Student’s t-test and Mann–Whitney U test were applied to evaluate differences between variables that followed normal and skewed distribution. Correlation analysis included Pearson parametric and Spearman non-parametric tests. Multiple linear regression analysis with enter model was used to assess the contribution of independent covariates to variations of the dependent variable. Observational study statistical results were provided by using the IBM^®^SPSS^®^ model 22.0 statistical package. *p* < 0.05 indicated statistically relevant differences between the observed data.

## 3. Results

**Figure 1:**
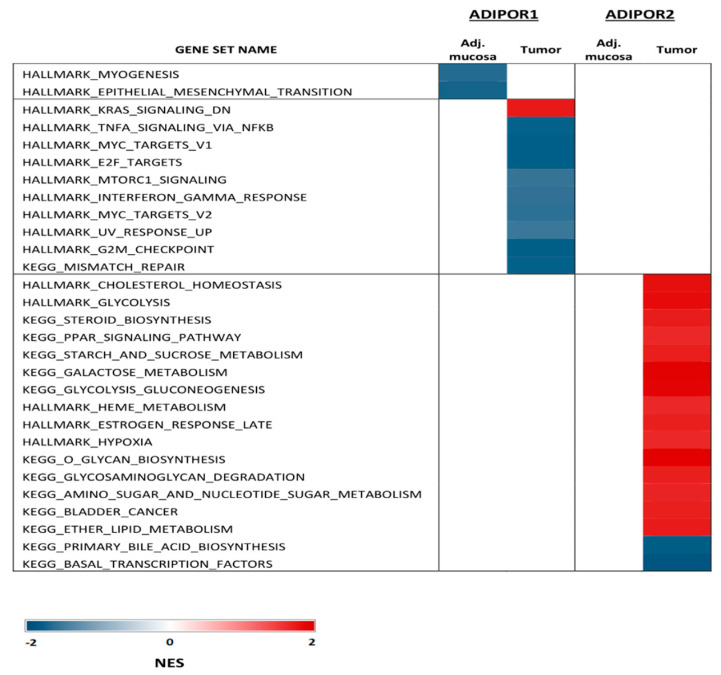
Significant signaling pathways associated with ADIPOR1 and ADIPOR2 mRNA levels in tumor tissue and healthy adjacent mucosa (GSEA of GSE 44076 dataset).

The GSEA analysis of the GSE44076 dataset showed that ADIPOR2 was positively correlated with metabolism-related gene sets, such as cholesterol homeostasis, glycolysis, steroid biosynthesis and the PPAR signaling pathway in tumor tissue (Supplementary File S1, Figure 1). Furthermore, in the same samples ADIPOR1 mRNA expression levels negatively correlated with the TNF-α NF-κB signaling pathway and mechanistic target of rapamycin kinase (MTORC1) (Supplementary File S1, Figure 1). This analysis also emphasized the negative correlation between ADIPOR1 gene expression and gene sets related to myogenesis and epithelial mesenchymal transition (EMT) in adjacent mucosa (Supplementary File S1, Figure 1).

**Figure 2:**
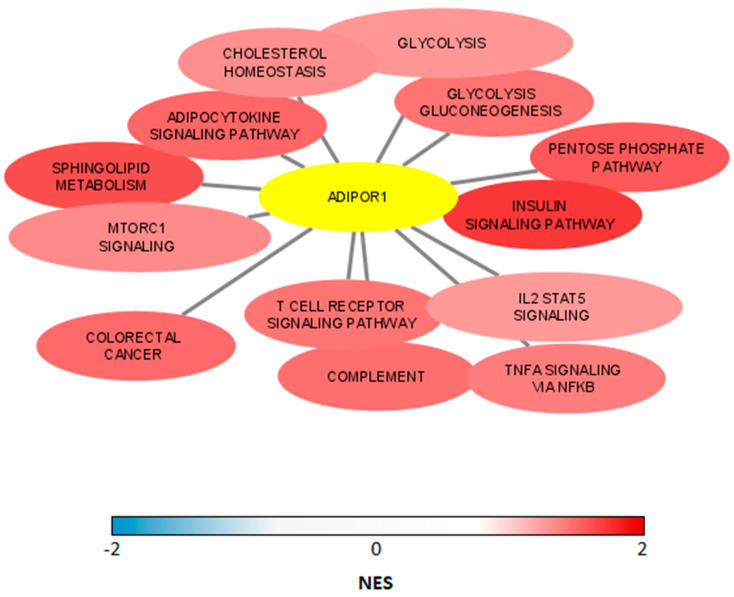
Several significant signaling pathways associated with ADIPOR1 mRNA levels in PBM (GSEA of GSE 47756 dataset).

On the contrary, the GSEA results of the GSE47756 dataset revealed a positive correlation between ADIPOR1 mRNA expression in the PBMs of CRC patients and gene sets related to metabolic pathways, such as cholesterol homeostasis, glycolysis and the insulin signaling pathway. Additionally, the TNF-α NF-κB signaling pathway and complement gene sets (Supplementary File S2, Figure 2) were enriched in cancer.

**Figure 3:**
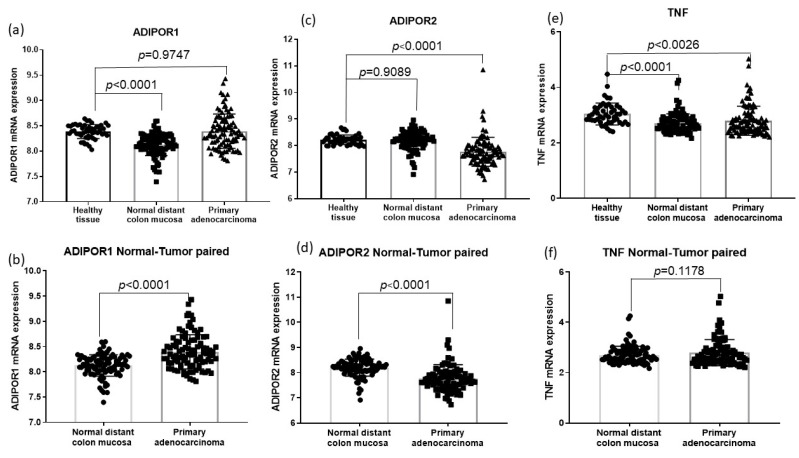
Differences in ADIPOR and TNF α mRNA levels among malignant tissue-adjacent mucosa and healthy tissue (GSE 44076 dataset). (**a**) Difference in ADIPOR1 mRNA levels between primary adenocarcinoma and healthy tissue and between adjacent mucosa and healthy tissue; (**c**) Difference in ADIPOR2 mRNA levels between primary adenocarcinoma and healthy tissue and between adjacent mucosa and healthy tissue; (**e**) Difference in TNF α mRNA between primary adenocarcinoma and healthy tissue and between adjacent mucosa and healthy tissue; (**b**) Difference in ADIPOR1 mRNA levels between primary adenocarcinoma and adjacent mucosa; (**d**) Difference in ADIPOR2 mRNA levels between primary adenocarcinoma and adjacent mucosa; (**f**) Difference in TNF α mRNA levels between primary adenocarcinoma and adjacent mucosa.

According to the GSE44076 dataset analysis, ADIPOR1 mRNA levels were increased in primary tumors when compared to adjacent normal colon mucosa, while ADIPOR2 mRNA levels were decreased in cancer (*p* < 0.0001; Figure 3b,d). There were no differences in TNF-α mRNA levels between these two groups (*p* = 0.1178, Figure 3f). Moreover, there was a significant difference in both ADIPOR1 and TNF-α expression levels between healthy and adjacent normal mucosa. ADIPOR1 and TNF-α expression levels were both decreased in tumor-adjacent normal cells compared to in healthy tissue (*p* < 0.0001; Figure 3a,e), while ADIPOR2 gene expression did not differ (*p* = 0.9089, Figure 3c). Furthermore, a comparison of ADIPOR and TNF-α mRNA levels between malignantly changed and healthy tissue revealed lower ADIPOR2 mRNA levels in cancer (*p* < 0.0001, Figure 3c) as well as lower TNF-α gene expression (*p* < 0.0026, Figure 3e), while ADIPOR1 levels did not differ between the groups (*p* = 0.9747, Figure 3a).

In the GSE47756 dataset, no significant differences in the PBM mRNA levels of ADIPOR1, ADIPOR2 and TNF-α were found between CRC and healthy cohorts (*p* = 0.1109; *p* = 0.3268; *p* = 0.6618, respectively).

In Table 1, we present the basic anthropometric characteristics and routine laboratory measurements of our two analyzed groups. Our cohorts were homogenous by gender distribution, while CRC patients were older and had lower body mass index (BMI) values (Table 1). Reduced TC, HDL-C and LDL-C levels were evident in the CRC group (Table 1). An evaluation of ADIPOQ’s receptor PBMC expression levels showed that ADIPOR1 mRNA levels were decreased in CRC, while ADIPOR2 mRNA levels did not differ between the groups (Table 1). A reduction in TNF-α mRNA levels was also noted in the CRC group (Table 1).

Next, we performed a correlation analysis to detect statistically significant associations between the studied variables (Table 2). In the CRC group, we recorded a positive correlation between TNF-α mRNA and HDL-C, while a negative association existed among HDL-C and ADIPOR1 mRNA levels (Table 2). In our healthy cohort, TNF-α and ADIPOR1 mRNA levels negatively correlated with TC, while a positive association between TNF-α and ADIPOR1 mRNA was noted (Table 2). Additionally, ADIPOR1 and ADIPOR2 mRNA levels positively correlated in both cohorts (Table 2).

There was no statistically significant difference in genotype distribution regarding ADIPOR1 SNP (rs7539542) between CRC patients and controls (*p* = 0.778). Thus, we sought differences in TNF-α, ADIPORs mRNA levels and lipid status parameters between genotypes of rs7539542 polymorphism. CRC patients with CG + GG genotype had lower concentrations of HDL-C, LDL-C and TC when compared to the CC genotype subgroup, while control subjects with identical genotypes had increased TG levels (Table 3). No significant differences in other examined parameters between subjects with CC and subjects with CG + GG genotypes, neither in CRC nor in the control group, were obtained (Table 3).

The same analyses were performed for ADIPOQ’s polymorphism (rs266729). We found no difference in genotype distribution between CRC patients and controls (*p* = 0.492). An analysis of differences across cohorts showed that CRC patients with CG and GG genotypes had lower TNF-α mRNA levels when compared to the CC genotype subgroup, albeit with borderline statistical significance (*p* = 0.053) (Table 4). In addition, the Hardy–Weinberg equilibrium was tested for both SNPs, and we found no deviations from it in either of the groups: ADIPOR1 rs7539542 (CRC: CC = 0.48, CG = 0.45, GG = 0.07; chi-square = 0.563; *p* = 0.453; control group: CC = 0.51, CG = 0.40, GG = 0.09; chi-square = 0.045; *p* = 0.832). ADIPOQ rs266729 (CRC: CC = 0.52, CG = 0.36, GG = 0.12; chi-square = 1.735; *p* = 0.188; control group: CC = 0.44, CG = 0.45, GG = 0.11; chi-square = 0.003; *p* = 0.955).

Based on the previously observed differences in parameters among genotypes (Table 3 and Table 4) as well as significant associations obtained via univariate regression analysis, we inspected the independent contribution of named SNPs to changes in lipid parameter levels (TC, HDL-C, LDL-C) as well as to the selected immune marker TNF-α mRNA by employing multivariate linear regression modeling. Model 1, which included ADIPOR1 rs7539542 and TG, explained 16.4% of variations in LDL-C levels, while model 2, with statistically significant covariates (ADIPOR1 rs7539542 and TG), explained 26.8% of variations in TC concentrations. In addition, a model including HDL-C (independent predictor of ADIPOR1 rs7539542 and TNF-α mRNA levels) provided statistical significance. However TNF-α gene expression alone contributed significantly to HDL-C changes; therefore, data regarding HDL-C were not herein presented. Finally, model 3, consisting of BMI and ADIPOQ rs266729, explained 9.9% of variations in TNF-α mRNA levels, with both variables representing independent and significant predictors of dependent variable changes. Complete model data are shown in Table 5.

## 4. Discussion

Despite many efforts, metabolic changes accompanying malignant disease development still need to be clarified. In this paper, we analyzed ADIPOR’s profile in patients with CRC. By using available bioinformatics datasets and by performing an observational research study, we demonstrated the associations between ADIPOQ homeostasis and energy metabolism parameters as well as ADIPOQ homeostasis’s association with TNF-α as an immune-based biomarker. Such an associational analysis of structurally and functionally related markers can lay the groundwork for PCR multiplex model development. This kind of multiple model analysis should preferably include readily available parameters. Namely, the discovery of reliable non-invasive blood biomarkers would increase patients’ adherence to screening programs, which is currently reduced due to the invasiveness and robustness of available screening and diagnostic tools. In order to adhere to this important principle of modern diagnostics, we used PBMCs as a source of genetic material.

Animal studies have already suggested the higher relevance of ADIPOQ’s protective effects mediated through ADIPOR1 compared to those exerted through the ADIPOR2 signaling cascade [29]. Our GSEA results for the GSE44076 dataset implied that ADIPOR2 was a more metabolically active receptor in malignancy, since the metabolism-related gene sets were associated with ADIPOR2, but not ADIPOR1 mRNA levels (Supplementary File S1, Figure 1). Nonetheless, ADIPOR1 mRNA levels were in negative correlation with gene sets related to pathways important for tumor progression, such as the TNF-α NFκB signaling pathway and MTORC, thus supporting the protective role of ADIPOR1 in CRC [30,31]. However, the MTOR cascade for the inhibition of CRC cell growth also depends on adenosine monophosphate-activated protein kinase (AMPK) activation, which further links cell proliferation with ADIPOQ’s metabolic response, making this interrelation even more complex [31]. Additionally, a negative correlation between ADIPOR1 mRNA levels and myogenesis and EMT in normal adjacent mucosa once again stressed the significance of ADIPOR1 as a mediator of ADIPOQ anti-neoplastic effects in affected tissues [32].

GSEA of another dataset (GSE47756) indicated that ADIPOR1 was an active receptor, since gene sets related to metabolic pathways and the TNF-α NF-κB signaling cascade were enriched (Supplementary File S2, Figure 2). This surprisingly positive association between ADIPOR1 and TNF-α was also demonstrated in our research, but only in the PBMCs of control participants (Table 2). Although the literature overview indicated the influence of TNF-α on ADIPOR1 mRNA levels [33,34], we found no such correlation in CRC (Table 2). Despite the obtained GSE47756 results, which were based solely on monocyte populations, it is reasonable to assume that other subpopulations, such as CD4 and CD8, B lymphocytes and NK cells, should also be taken into account before deriving any final conclusions regarding ADIPOR1-TNF-α associational analysis, as well as its expression levels. Additionally, ample evidence supports ADIPOQ’s immune response effects conveyed through these subpopulations [5,10,35]. Therefore, PBMCs should be considered for future research in this area to gain an overall insight regarding the role of ADIPOQ in the immune response.

Considering that our in silico analysis repeatedly emphasized ADIPORs’ relations with TNF-α, we selected TNF-α as a relevant marker of the immune system. Therefore, we included it in the case–control part of the research. Lower levels of PBMC TNF-α mRNA were found in our CRC cohort (Table 1). A reduction in TNF-α mRNA levels was also observed in the GSE44076 dataset between malignantly transformed tissue and control subjects’ healthy mucosa and between tumor-adjacent and healthy mucosa. Some authors [36] found an association between higher colon cancer survival rate and increased TNF-α expression in tumor-infiltrating lymphocytes. By contrast, others [37] implied elevated TNF-α mRNA levels in the malignant tissue samples of subjects with progressive stages of CRC. In addition to those equivocal reports, Ganapathi et al. reported that the downregulation of TNF-α in the PBMCs of CRC patients occurs due to microsatellite instability [38].

Our research further suggested the association of ADIPOQ gene polymorphism, rather than ADIPOR1, with maintaining TNF-α homeostasis. Considering that the relationship between ADIPOR1 and TNF-α was lacking in our patients, we chose ADIPOQ’s polymorphism as the second polymorphism of interest to inspect its long-known relationship with TNF-α in CRC. Namely, the association of ADIPOQ’s polymorphism rs266729 with TNF-α mRNA levels was recorded in the cancer group (Table 4). Previously, this SNP was linked to a lower CRC development risk [39]. Lower TNF-α mRNA levels were found in CRC patients with CG and GG genotypes (*p* = 0.053). This relationship was not found in our control group. We further conducted multiple linear regression analyses to explore the observed ADIPOQ–TNF-α mRNA association. ADIPOQ polymorphism and BMI explained 9.9% of the variation in TNF-α mRNA levels (Table 5). Divella et al. previously reported that CRC patients with rs266729 CG and GG genotypes had higher circulating TNF-α while having lower ADIPOQ concentrations [40]. Therefore, these results confirm the relationship between rs266729 polymorphism and immune homeostasis reflected through changes in both protein and transcript levels of TNF-α. However, inconsistencies in our obtained conclusion have to be noted.

Being located in the 3′ untranslated region of the ADIPOR1 gene, ADIPOR1 rs7539542 and precisely its GG genotype was reportedly related to a 30−40% reduction in PBMC ADIPOR1 mRNA levels [41]. However, our results did not confirm such findings (Table 3). Similarly, we did not find any association between the observed polymorphism and TNF-α mRNA levels (Table 3). Nevertheless, it should be emphasized that our group of CRC patients with CG + GG genotypes had lower TC, LDL-C and HDL-C levels than their counterparts with the CC genotype (Table 3). The association of this SNP with serum lipids was also evident in our healthy cohort, since higher TG levels were observed in the CG + GG genotype group (Table 3). Furthermore, the results of a multiple linear regression analysis demonstrated that a model consisting of TG concentration and rs7539542 explained 16.4% of the variation in LDL-C levels, while a model comprising the same independent variables explained 26.8% of variations in TC levels. The Diabetes Heart Study has already shown the association of rs7539542 with TC levels, but in subclinical cardiovascular diseases, whereas this association was scarcely reported in cancer [42]. Although several studies demonstrated a relationship between ADIPOR1 polymorphism and lipid homeostasis disturbances [42,43], the mechanisms are yet to be discovered. One of the proposed explanations is based on the location of rs7539542 in the 3′ untranslated gene region and suggests this genetic variant may act as a destabilizing element of ADIPOR1 mRNA. However, this was not the case in our cohort. We did not find a relationship between rs7539542 genotypes and ADIPOR1 mRNA levels. This polymorphism may have influenced phenotype traits by interacting with other SNPs not investigated here.

On the one hand, we observed a discrepancy regarding the lack of differences in the distribution of ADIPOR1 genotypes among the CRC and control groups. On the other hand, we also observed an association of this SNP with markedly changed lipid status parameters in our two cohorts. These two observations warrant further attention. The relatively small sample size for our SNP analysis could be responsible for the lack of observed significant differences in this genetic trait. Moreover, it should be noted that reduced concentrations of lipid status parameters might arise because of cachexia–anorexia syndrome occurring in the advanced stage of CRC. Thus, the observed association of lipid markers with ADIPOR1 might be largely indirect. It is noteworthy that ADIPOQ suppresses colon cancer cell growth by inhibiting lipogenic gene expression due to AMPK activation [44]. Such findings confirm the involvement of the ADIPOQ/ADIPOR axis in maintaining lipid homeostasis. Finally, our correlation analysis (Table 2) showed a negative association between HDL-C concentrations and ADIPOR1 mRNA levels. However, all named conclusions should be verified by prospective studies with a larger sample size.

Regarding the genetic profiles of ADIPORs, we observed lower levels of PBMC ADIPOR1 mRNA in CRC compared to in controls, while similar expression levels of ADIPOR2 existed in our cohorts (Table 1). Surprisingly, our research showed distinctive ADIPOR gene expression patterns. A study by Van Stijn et al. implicated that ADIPOQ itself can upregulate its receptors’ mRNA levels in THP−1 monocytes, with a more significant influence exhibited in ADIPOR2 than in ADIPOR1 [45]. Considering previously reported alterations to ADIPOQ levels in CRC patients [46,47], our results might reflect ADIPOQ’s involvement in the imbalance of its receptors’ gene expressions. It was also observed that the downregulation of LXR and PPARγ signaling pathways in M1 macrophages leads to a reduction in ADIPOR levels, suggesting that macrophage polarization alters ADIPOR expression and, consequently, the ADIPOQ-mediated inflammatory response [45]. Dalamaga et al. suggested that the omnipresence of both receptors in different tissues can be expected, but one form usually prevails [9].

Furthermore, our experimental PBMC results were in accordance with previous findings on reduced ADIPOR1 expression levels in patients with advanced colorectal malignancy, such as lymph node invading tumors [48]. Namely, most of our patients (44%) were in the progressive stage of the disease according to the Astler–Coller classification system. In addition, Hiyoshi et al. pointed to the interesting hypothesis that ADIPOR downregulation represents a mechanism used by malignant cells to counteract ADIPOQ-mediated anti-neoplastic effects, especially in lymph node metastasis [48]. Moreover, considering that higher ADIPOR1 expression in the macrophages of transgenic mice was related to lower proinflammatory cytokine levels, we assume that reduced PBMC ADIPOR1 mRNA levels in our CRC patients could result in higher exposure to an enhanced and prolonged inflammatory state [49].

Our in silico analysis of the GSE44076 dataset also implied variable ADIPOR gene expressions. Namely, when adjacent mucosa was compared to malignantly transformed mucosa, decreased ADIPOR2 mRNA and increased ADIPOR1 mRNA levels were demonstrated in tumor tissue. Moreover, when the tissue of healthy subjects was compared to CRC, decreased ADIPOR2 mRNA levels were again obtained in the tumor, whereas ADIPOR1 gene expression did not differ. Analogous to our results on PBMCs (Table 1) when tumor-adjacent mucosa was compared to the healthy mucosa, lower ADIPOR1 mRNA levels were obtained in adjacent mucosa, while ADIPOR2 mRNA levels did not differ. Based on the aforesaid results, determining ADIPORs’ gene expression patterns might be helpful for CRC patients. Furthermore, evaluating ADIPORs’ gene expressions in PBMCs might become part of innovative PCR multiplex testing panels for monitoring immunomodulatory therapy.

In this research, we were guided by the idea of expanding the current knowledge on the role of ADIPOQ in CRC development, not only through its participation within the AMPK signaling cascade, but also through its genetic influences on lipid homeostasis. Given the complexity of cancer development, novel indicators that encompass and depict multiple pathological processes are needed. In line with this, ADIPORs, as mediators of ADIPOQ’s effects, might represent integrative biomarkers that reflect disturbances in multiple homeostatic mechanisms, in the first place, hormonal, metabolic, immune and inflammatory mechanisms. Although ADIPORs’ mRNA levels have already been thoroughly analyzed in cancer tissues, herein, we opted for PBMCs as alternative biological samples. Modern laboratory diagnostics tend towards a prevailing use of readily accessible biological samples that should ensure the steady quality of laboratory results in parallel with enhanced patient comfort and increased adherence to screening programs. Having this in mind, PBMCs might be very useful and reliable yet easily obtainable specimens for liquid biopsy.

## 5. Conclusions

In summary, our GSEA analysis implicated the more prominent role of ADIPOR2 in the metabolic alterations of malignant tissue. At the same time, the protective function of ADIPOR1 in tumor progression was indicated through its negative association with EMT and myogenesis in adjacent mucosa. An in silico analysis of the GSE44076 dataset showed that tumor ADIPOR1 mRNA expression was unchanged compared to the healthy tissue, while its downregulation was recorded in tumor-adjacent mucosa. By contrast, ADIPOR2 mRNA levels in tumor-adjacent mucosa were equivalent to the levels in healthy cells while being decreased in tumors. Additionally, our case–control study’s finding of downregulated ADIPOR1 gene expression and unaltered ADIPOR2 in PBMCs was equivalent to the transcriptional trend observed in tumor-adjacent mucosa. PBMC ADIPOR1 downregulation again led us to question its plausible association with tumor progressive mechanisms, but now at the level of immune cells. In addition, our in silico findings revealed decreased TNF-α mRNA levels in tumor adjacent mucosa and tumor in comparison to healthy tissue levels, which was in congregation with decreased TNF-α mRNA levels obtained in PBMCs.

Moreover, ADIPOR1 was singled out as a metabolically active receptor in monocytes (GSE44756) while at the same time being associated with TNF-α NF-κB gene set enrichment. Similarly, a positive association between ADIPOR1 and TNF-α gene expressions in PBMCs was found in our case–control study. Findings such as these support previous claims of ADIPOQ’s dual nature during inflammatory response development with TNF-α as one of the primary mediators of ADIPOQ’s immunomodulatory effects. Finally, our case–control observations of lower ADIPOR1 and TNF-α mRNA in the PBMCs of CRC patients in contrast to in silico non-existing differences in PBMs suggest that apart from monocytes, other subpopulations of white blood cells should be evaluated as well. Our SNP analysis implicated that ADIPOQ homeostasis was associated with an altered immune response and metabolic disturbances, observed as lower levels of TNF-α mRNA and lower TC, LDL-C and HDL-C concentrations in the G allelic carriers of both ADIPOQ and ADIPOR1 SNP, respectively. Therefore, the results of our study might contribute to an improved understanding of two delicate and closely related homeostases: ADIPOQ and TNF-α. The obtained conclusions shed light on ADIPORs as essential components of immunity and energy metabolism and whose dysregulation could favor peritumoral milieu formation.

## Figures and Tables

**Table 1:** General anthropometric and laboratory data of CRC patients and control group.

Parameter	CRC Patients (N = 73)	Control Group (N = 80)	*p*
Gender (f/m)	24/49	38/42	0.066
Age (years) ^a^	66.5 (58–74)	53 (50.2–58)	<0.001
BMI (kg/m^2^)	24.695 ± 2.925	26.202 ± 3.984	<0.050
TC (mmol/L) ^a^	4.438 (3.700–5.025)	5.669 (4.951–6.388)	<0.001
HDL-C (mmol/L) ^b^	0.985 (0.910–1.066)	1.269 (1.165–1.382)	<0.001
LDL-C (mmol/L) ^a^	2.890 (2.130–3.266)	3.682 (2.869–4.372)	<0.001
TG (mmol/L) ^b^	1.241 (1.152–1.336)	1.244 (1.133–1.366)	0.964
ADIPOR-1 mRNA levels ^a^	0.535 (0.446–0.748)	0.849 (0.706–1.088)	<0.001
ADIPOR-2 mRNA levels ^b^	0.978 (0.913–1.047)	0.939 (0.869–1.016)	0.442
TNFα mRNA levels ^b^	0.792 (0.688–0.912)	0.962 (0.877–1.056)	<0.050

Data are presented as mean ± standard deviation and compared by Student’s *t*-test. ^a^ Data are presented as median with interquartile range. Comparison was performed by Mann–Whitney *U* test. ^b^ Data are presented as geometrical mean (95% confidence interval for mean).

**Table 2:** Significant correlations of lipid status parameters and ADIPOR1, ADIPOR2 and TNF α mRNA levels in CRC and control group.

	CRC Patients	
Parameter	HDL-C (mmol/L)	Normalized ADIPOR1 mRNA
TNF α mRNA levels	r = 0.238; *p* < 0.05	ρ = 0.142; *p* = 0.230
ADIPOR2 mRNA levels	r = −0.129; *p* = 0.278	ρ = 0.268; *p* < 0.05
ADIPOR1 mRNA levels	ρ = −0.262; *p* < 0.05	/
	Control group	
Parameter	TC (mmol/L)	Normalized ADIPOR1 mRNA
TNF α mRNA levels	ρ = −0.228; *p* < 0.05	r = 0.619; *p* < 0.001
ADIPOR2 mRNA levels	ρ = −0.204; *p* = 0.072	r = 0.634; *p* < 0.001
ADIPOR1 mRNA levels	ρ = −0.230; *p* < 0.05	/

**Table 3:** Differences in the examined parameters in relation to SNP ADIPOR1 (rs7539542).

Parameter	CRC			Control Group		
	CC	CG + GG	*p*	CC	CG + GG	*p*
BMI	25.279 ± 2.606	24.073 ± 3.153	*p* = 0.094	26.030 ± 3.546	26.383 ± 4.439	*p* = 0.698
TG (mmol/L)	1.275 (1.167–1.391)	1.210 (1.073–1.365)	*p* = 0.489 ^b^	1.098 (0.965–1.251)	1.413 (1.244–1.605)	*p* = 0.006 ^b^
HDL-C (mmol/L)	1.140 ± 0.437	0.952 ± 0.241	*p* = 0.029	1.276 (1.111–1.464)	1.262 (1.136–1.403)	*p* = 0.904 ^b^
TC (mmol/L)	4.839 (4.472–5.237)	3.963 (3.648–4.306)	*p* = 0.001 ^b^	5.601 ± 1.037	5.668 ± 1.056	*p* = 0.777
LDL-C (mmol/L)	3.068 (2.751–3.423)	2.401 (2.114–2.727)	*p* = 0.004 ^b^	3.663 ± 0.963	3.664 ± 1.013	*p* = 0.996
ADIPOR1 mRNA levels	0.535 (0.454–0.674)	0.528 (0.399–0.794)	*p* = 0.453 ^a^	0.870 (0.691–1.096)	0.834 (0.725–1.073)	*p* = 0.866 ^a^
ADIPOR2 mRNA levels	0.979 (0.885–1.085)	0.977 (0.887–1.075)	*p* = 0.961 ^b^	0.792 (0.668–1.192)	0.939 (0.776–1.145)	*p* = 0.220 ^a^
TNF-α mRNA levels	0.837 (0.647–1.219)	0.794 (0.579–1.204)	*p* = 0.651 ^a^	0.954 (0.825–1.104)	0.971 (0.862–1.093)	*p* = 0.856 ^b^

Data are presented as mean ± standard deviation and compared by Student’s *t*-test. ^a^ Data are presented as median with interquartile range. Comparison was performed by Mann–Whitney U test. ^b^ Data are presented as geometrical mean (95% confidence interval for mean).

**Table 4:** Differences in the examined parameters in relation to SNP ADIPOQ (rs266729).

Parameter	CRC			Control Group		
	CC	CG + GG	*p*	CC	CG + GG	*p*
BMI	24.570 ± 3.201	24.826 ± 2.646	*p* = 0.725	26.254 ± 3.802	26.159 ± 4.170	*p* = 0.917
TG (mmol/L)	1.284 (1.168–1.413)	1.195 (1.061–1.345)	*p* = 0.335 ^b^	1.272 (1.119–1.444)	1.223 (1.067–1.403)	*p* = 0.685 ^b^
HDL-C (mmol/L)	0.995 (0.883–1.120)	0.975 (0.875–1.086)	*p* = 0.803 ^b^	1.276 (1.126–1.446)	1.264 (1.120–1.425)	*p* = 0.910 ^b^
TC (mmol/L)	4.454 (4.058–4.889)	4.263 (3.934–4.619)	*p* = 0.475 ^b^	5.808 ± 1.047	5.503 ± 1.028	*p* = 0.199
LDL-C (mmol/L)	2.786 (2.456–3.160)	2.611 (2.302–2.961)	*p* = 0.463 ^b^	3.828 ± 0.960	3.539 ± 0.989	*p* = 0.196
ADIPOR1 mRNA levels	0.526 (0.448–0.646)	0.549 (0.401–0.885)	*p* = 0.903 ^a^	0.802 (0.661–1.100)	0.876 (0.728–1.083)	*p* = 0.385 ^a^
ADIPOR2 mRNA levels	1.024 (0.923–1.137)	0.931 (0.852–1.017)	*p* = 0.166 ^b^	0.885 (0.729–1.145)	0.872 (0.679–1.192)	*p* = 0.812 ^a^
TNF α mRNA levels	0.939 (0.680–1.302)	0.704 (0.468–1.171)	*p* = 0.053 ^a^	0.987 (0.643–1.393)	0.977 (0.776–1.163)	*p* = 0.687 ^a^

Data are presented as mean ± standard deviation and compared by Student’s *t*-test. ^a^ Data are presented as median with interquartile range. Comparison was performed by Mann–Whitney U test. ^b^ Data are presented as geometrical mean (95% confidence interval for mean).

**Table 5:** Multiple linear regression models of patients with CRC.

Model 1	Standardized Beta (Standard Error)	*p* Value	Adjusted R-Square and Model’s *p* Value
TG (mmol/L)	0.281 (0.127)	0.011	R^2^ = 0.164; *p* = 0.001
SNP *ADIPOR1*	−0.307 (0.035)	0.006	
Model 2	Standardized beta (standard error)	*p* value	Adjusted R-square and model’s *p* value
TG (mmol/L)	0.375 (0.083)	0.000	R^2^ = 0.268; *p* = 0.000
SNP *ADIPOR1*	−0.355 (0.023)	0.001	
Model 3	Standardized beta (standard error)	*p* value	Adjusted R-square and model’s *p* value
BMI (kg/m^2^)	0.280 (0.605)	0.021	R^2^ = 0.099; *p* = 0.014
SNP *ADIPOQ*	−0.237 (0.062)	0.049	

Model 1: Dependent variable: LDL-C concentration. Model 2: Dependent variable: TC concentration. Model 3: Dependent variable: TNF α mRNA levels. Independent and dependent continuous variables were log-normalized.

## Data Availability

The datasets used and/or analyzed during the current study are available from the corresponding author on reasonable request.

## Supplementary Materials

The following supporting information can be downloaded at: https://www.mdpi.com/article/10.3390/ijerph192214995/s1, File S1: GSE44076 GSEA reports; File S2: GSE47756 GSEA reports.
